# Molecular epidemiology of *Bordetella pertussis* in Greece, 2010–2015

**DOI:** 10.1099/jmm.0.000688

**Published:** 2018-02-02

**Authors:** Evangelia Petridou, Christel Barker Jensen, Athanasios Arvanitidis, Maria Giannaki-Psinaki, Athanasios Michos, Karen Angeliki Krogfelt, Randi Føns Petersen

**Affiliations:** ^1^​ Department of Clinical Microbiology, ‘Aghia Sophia’ Children's Hospital, Athens, Greece; ^2^​ Department of Virus and Microbiological Special Diagnostics, Parasites & Fungi, Statens Serum Institut, Copenhagen, Denmark; ^3^​ Department of Bacteria, Parasites & Fungi, Statens Serum Institut, Copenhagen, Denmark; ^4^​ First Department of Pediatrics, National and Kapodistrian University of Athens, ‘Aghia Sophia’ Children's Hospital, Athens, Greece

**Keywords:** whooping cough, *Bordetella pertussis*, molecular typing, multi-Locus variable number tandem repeat analysis (MLVA), sequence-based typing, Greece

## Abstract

**Purpose:**

To determine the predominant strains of *Bordetella pertussis* in Greece during 2010–2015.

**Methodology:**

Infants and children (*n*=1150) (15 days to 14 years) of Greek, Roma and immigrant origin with different vaccination statuses were hospitalized in Athens, Greece with suspected pertussis infection. IS*481*/IS*1001* real-time PCR confirmed *Bordetella* spp./*B. pertussis* infection in 300 samples. A subset of samples (*n*=153) were analysed by multi-locus variable number tandem repeat analysis (MLVA) and (*n*=25) by sequence-based typing of the toxin promotor region (*ptxP*) on DNA extracted from clinical specimens.

**Results/Key findings:**

A complete MLVA profile was determined in 66 out of 153 samples; the *B. pertussis* MLVA type 27 (*n*=55) was the dominant genotype and all tested samples (*n*=25) expressed the *ptxP3* genotype. The vaccine coverage in the Greek population was 90 %; however, the study population expressed complete coverage in 2 out of 264 infants (0–11 months) and in 20 out of 36 children (1–14 years). Roma and immigrant minorities represent 7 % of the Greek population, but make up 50 % of the study population, indicating a low vaccine coverage among these groups.

**Conclusions:**

The *B. pertussis* MT27 and *ptxP3* genotype is dominant in Greek, Roma and immigrant infants and children hospitalized in Greece. Thus, the predominant MLVA genotype in Greece is similar to other countries using acellular vaccines.

## Introduction

Whooping cough (pertussis) is a highly contagious respiratory infection caused mainly by the bacterium *Bordetella pertussis* (*B. pertussis*). The introduction of pertussis vaccination during the 1950s and 1960s resulted in a dramatic reduction (>90 %) in the pertussis incidence and mortality in the industrialized world. However, this vaccine-preventable disease continues to circulate in many countries [1]. Consequently, it remains a disease with high morbidity and mortality especially in infants who have not been fully immunized [2, 3]. Like in many industrialized countries, a national immunization programme (NIP) targeting *B. pertussis* was initiated in Greece more than 50 years ago [3, 4]. A whole-cell vaccine was introduced in 1951, which was then replaced with an acellular counterpart in 1997 [3]. Currently, two commercial acellular pertussis vaccines are licensed for immunization on the Greek market administered in combination with diphtheria, tetanus, acellular pertussis, inactivated poliomyelitis and *Haemophilus influenzae* type b conjugate vaccines (DTaP/IPV/Hib). The NIP includes mandatory immunization against *B. pertussis* at ages 2, 4 and 6 months (DTaP) [5]. Two booster doses are offered to children at 15–18 months and 4–6 years of age (DTaP) [5]. Finally, adolescents 11–18 years of age (preferably at the age of 11–12 years) receive a single dose of a combination vaccine containing acellular components of *B. pertussis* together with diphtheria and tetanus toxoids (Tdap) [5]. Regarding the first five DTaP doses, studies from 2006 and 2012 showed a 90 % vaccination coverage in the Greek population [6]. Despite the relatively high vaccination coverage, pertussis remains a public health concern in the Greek population [7]. The Greek population comprises 93 % native Greek citizens and 7 % foreign citizens of which approximately 2.5 % (300 000) are Roma [8]. According to data for the period 2004–2014 published by the Hellenic Center for Disease Control and Prevention, Ministry of Health, Greece, the disease appears to affect all ages, but it presents the highest frequency of occurrence in the age group 0–4 years (especially among infants below 1 year of age) [7]. The age group >45 is less represented among notified/laboratory confirmed pertussis cases [7], which is likely to be explained by underreporting compared to a true absence of disease in this group. The reason for the lack of immunization in infants and children is that they are either too young to be vaccinated or that they have not been enrolled in the NIP [9]. The incidence of pertussis is also higher in children with Roma background [9]. Still, these unvaccinated cases cannot explain the continuing circulation of *B. pertussis* observed in Greece and in other industrialized countries [10, 11]. Recurring epidemics are attributed to factors such as improved diagnostics, increased awareness, waning immunity and pathogen adaptation [12].

Molecular typing of *B. pertussis* strains is important in order to monitor the spread of virulent strains and emerging vaccine-escape variants. Typically, molecular typing is performed on cultured isolates from patients suffering from pertussis [13]. *B. pertussis* is a fastidious, slow-growing bacterium requiring suitable nasopharyngeal specimens to be taken close to onset of disease, timely transport to the laboratory and specialized media to increase the likelihood of successful recovery.

Many routine diagnostic laboratories use PCR as the gold standard method in early diagnosis since the technique produces a more rapid result and has a higher sensitivity compared to culturing. With the increased use of *B. pertussis* PCR in diagnostic laboratories, the number of laboratories attempting to isolate *B. pertussis* has decreased. Consequently, there are fewer isolates available to send to reference laboratories for further characterization. The typing of circulating *B. pertussis* strains thus relies on the DNA present in nasopharyngeal specimens. Multi-locus variable number tandem repeat analysis (MLVA) was applied to provide a molecular fingerprint of *B. pertussis* in Greece. In addition, sequencing of the pertussis toxin promoter gene *ptxP* was performed on a subset of MLVA-typed samples.

MLVA examines highly polymorphic regions containing variable numbers of tandem repeats (VNTRs) in six different loci as described by Schouls *et al.* [14]. Currently (September 2017), 333 distinct MLVA types (MTs) have been detected worldwide according to the international *B. pertussis* MLVA reference database [15].

In Denmark, genetic typing of *B. pertussis* strains from 1949 to 2010 showed that before the introduction of pertussis vaccination, the strain diversity was higher compared to that seen following the introduction of the vaccination programme [16]. During the non-vaccine period, a more even distribution among the major MTs was observed. The whole-cell and acellular vaccine time periods correlated with both the presence of fewer MTs and the dominance of single MTs. MT27 was found to predominate in countries using acellular pertussis vaccines, including European [17, 18] and non-European countries, such as Australia [19], Japan [20] and the USA [21]. In countries currently using a whole-cell vaccine, such as Poland [22] and the Philippines [23], MTs other than MT27 are predominant. In Poland, MT29 (27 %) and MT70 (16 %) were found to predominate during the 54-year test period (1959–2013) [22]. In the Philippines, MT34 (50 %) (2012–2014) – a rare MT in Europe, Australia, Japan and the USA – was predominant [23]. In China (2012–2013), a combination of whole-cell and acellular vaccines was in use, and MT55 (52 %) and MT104 (13 %) were prevalent, whereas MT27 was only present in 6 % [24].

The purpose of this study was to determine the predominant strains of *B. pertussis* circulating in Greece during 2010–2015. This is the first molecular typing study of *B. pertussis* conducted on samples from infants and children hospitalized in Greece.

## Methods

### Study population and clinical specimens

All infants and children included in the study were hospitalized and (according to the clinical presentation and national guidelines) suspected of having pertussis. Nasopharyngeal aspirates from 1150 infants and children (aged from 15 days to 14 years) were tested for pertussis during the period from 2010 to 2015 at the Department of Clinical Microbiology of the ‘Aghia Sophia’ Children’s Hospital, Athens, which is the larger tertiary paediatric hospital in Greece. The department hosts the only public laboratory for molecular diagnosis of pertussis in Greece. Culturing was not performed.

### Detection of *Bordetella* spp./*B. pertussis* DNA by real-time PCR

DNA from 200 µl sample was extracted using a MagNA Pure Compact Instrument in combination with the MagNA Pure Compact Nucleic Acid Isolation Kit I (Roche, Basel, Switzerland). All samples were eluted into a final volume of 50 µl of the MagNA Pure elution buffer. Real-time PCR amplification of *Bordetella* DNA was performed in duplicates in 50 µL-reaction volumes with the addition of 5 µl undiluted DNA and 5 µl1 : 10 dilution of DNA. PCR was performed according to the manufacturer’s instructions using the LightMix Kit *Bordetella pertussis* and *parapertussis* (TIB MolBiol, Berlin, Germany) using a LightCycler 2.0 Instrument (Roche) with LightCycler 4.1 software. An internal PCR control provided by the manufacturer was included in the reaction mix to avoid false-negative reporting. The kit targets the IS*481* and the IS*1001* insertion sequences. Interpretation of results was as follows: samples positive for IS*481* were scored as *B. pertussis*/*B. holmesii*/*B. bronchiseptica*, although considered most likely to be *B. pertussis;* those positive for IS*1001*, were scored as *B. parapertussis*/*B. bronchiseptica*; those positive for both IS*481* and IS*1001* were scored as *B. bronchiseptica*; and those negative for either target as *Bordetella* spp. not detected. All IS*481* positive samples were tested for the presence of *B. holmesii* using primers and probes designed and provided by TIB MolBiol (BHIS_F 5′-ggTgTTgAgCCggTggC-3′, BHIS_S 5′-gTTgA gCCggTggCgAC-3′, BHIS_A 5′CgCCgCCTTggCTCAC-3′, BHIS_R 5′-CATCgCCgCCTTggC-3′, BHIS_FL 5′-TCgCg CTCAAgCTAAAAgCCTATC-FL-3′). Purified DNA was stored at −20 °C.

### Molecular typing by multi-locus variable number tandem repeat analysis (MLVA)

Samples which tested positive for the IS*481* element and negative for IS*1001*, i.e. most likely consistent with the presence of DNA from *B. pertussis*, were randomly selected by year and sent to Denmark for MLVA typing (in total 153 samples, Table 1).

**Table 1.** Nasopharyngeal aspirates tested IS*481* positive by PCR at ‘Aghia Sophia’ Children’s Hospital, Athens, Greece (2010–2015)

Year	Samples tested	IS*481* positive	Positive samples selected for typing
2010	297	84 (28 %)	27
2011	143	28 (20 %)	21
2012	166	44 (27 %)	30
2013	190	55 (29 %)	37
2014	210	51 (24 %)	27
2015	144	38 (26 %)	11
**Total**	**1150**	**300**	**153**

The molecular typing was carried out at the Department of Virus and Microbiological Special Diagnostics, Statens Serum Institut (SSI), Copenhagen, Denmark. Typing was performed on all samples received from Greece. If a sample did not give a complete MT, the sample was tested with SSI in-house diagnostic PCR. The SSI PCR is a conventional assay where screening for *Bordetella* spp. DNA is performed using a multiplex IS*481*/IS*1001* PCR modified from Glare *et al.* [25] and van der Zee *et al.* [26], and confirmation of *B. pertussis* DNA is performed using a *ptxP* PCR modified from Birkebæk *et al.* [27]. The SSI PCR is performed using Immolase DNA polymerase (Bioline, UK) in 100 µl reaction volumes with the addition of 10 µl DNA on a GeneAmpPCR System 9700 thermocycler (Applied Biosystems, Waltham, MA, USA).

The number of tandem repeats in six loci (VNTR1, VNTR3a, VNTR3b, VNTR4, VNTR5 and VNTR6) was determined using a method originally described by Schouls *et al*. [14]. This method was optimized for the typing of clinical specimens by Litt *et al*. [13]. The PCR protocol used was modified from previously published protocols [13, 14, 16, 28]. Monoplex amplification was performed in a total volume of 20 µl containing 10 µl HotStarTaq Master Mix (Qiagen, Hilden, Germany), 4 µl betaine (5 M) (Sigma-Aldrich, St. Louis, MO, USA) for VNTR1, 3, 4, and 5, or 6 µl betaine (5 M) for VNTR6, 1 µl of each primer (10 pmol µl^−1^), and 1 or 2 µl DNA. The thermal cycling parameters included an initial denaturation step at 95 °C for 15 min, followed by 40 cycles of 95 °C for 30 s, 60 °C for 30 s, and 72 °C for 90 s, and a final extension step of 72 °C for 10 min. PCR was run on a GeneAmpPCR System 9700 thermocycler (Applied Biosystems). The primers used for MLVA are presented in Schouls *et al.* [14].

Additional PCR amplification was applied to samples giving an incomplete PCR result (when a PCR product was obtained in less than the normal numbers of loci (5/6 loci as VNTR3b is a duplication of VNTR3a and rarely seen) using the same primers and conditions as described for monoplex PCR.

The amplicon size of the PCR products was determined on an ABI 3130 Genetic Analyzer with GeneScan-600LIZ (Applied Biosystems) as an internal lane size standard, or using a Fragment Analyzer (Advanced Analytical Technologies, Ankeny, IA, USA) with the dsDNA 905 Reagent Kit (Advanced Analytical Technologies).

### Data analysis for MLVA

The size of each amplicon determined by capillary electrophoresis was converted into the number of repeats using a custom-made conversion table. Each sample was assigned a row of numbers representing the number of repeats at each VNTR allele in the order: VNTR1, 3a, 3b, 4, 5 and 6. All unique combinations of integers were assigned a MT according to the international *B. pertussis* MLVA reference database (www.mlva.net/bpertussis/default.asp) [15]. Novel MTs were assigned by Dr H. G. J. van der Heide (National Institute for Public Health and the Environment, Bilthoven, the Netherlands).

To verify the conversion of amplicon size obtained by capillary electrophoresis to the number of repeats, one to three representatives of different amplicon sizes for each VNTR region were sequenced (Macrogen, Amsterdam, the Netherlands). The sequencing results showed that the amplicon size obtained by capillary electrophoresis diverged from the size obtained by sequencing. The difference in size was stable for all investigated sizes within each VNTR region. Therefore, a correction factor was introduced to calculate the correct number of repeats. The discrepancy between the estimated size and the sequenced size of the amplicons may be due to the formation of secondary structures [14]. DNA sequencing was used to verify the number of repeats in cases of unclear analysis. The *B. pertussis* Tohama I strain (ATCC BAA-589) and the type strain (ATCC 9797^T^) were included as controls and new MTs were verified by sequencing (Macrogen, Amsterdam, the Netherlands).

The MLVA profiles were clustered in the BioNumerics software (version 7.6) (Applied Maths, Sint-Martens-Latem, Belgium) using a categorical coefficient and visualized by the minimum spanning tree method. The genetic diversity (Simpson’s index of diversity) was calculated by means of the online tool Comparing Partitions (www.comparingpartitions.info) [29] for quantitative assessment of partition congruence. The value of this index ranges from 0 to 1 and greater values indicate greater diversity.

### Pertussis toxin promoter gene (*ptxP*) typing

Twenty-five samples generating a complete MLVA profile were selected for sequence-based typing of the potentially polymorphic pertussis toxin promoter (*ptxP*) using the primers described by Schouls *et al*. [14]. The presence of *B. pertussis* DNA in all selected samples were confirmed by the SSI *ptxP* PCR. The selection was made to include samples from infants and children of different origin as well as those from which varying MTs including the predominant MT were found.

## Results

### Detection of *Bordetella* spp. in clinical samples

In total, 1150 infants and children ranging in age from 15 days to 14 years, clinically suspected of having a pertussis infection, were hospitalized during the period 2010–2015. Laboratory PCR confirmation was performed by detection of DNA in nasopharyngeal aspirates.

*B. parapertussis/B. bronchiseptica* were detected in 32 of 1150 infants and children (2.8 %) by IS*1001* PCR. *B. pertussis/B. holmesii/B. bronchiseptica* was detected in 300 of 1150 samples by IS*481* PCR. The presence of *B. holmesii* could not be confirmed by a species-specific PCR and neither were any samples positive for both IS*1001* and IS*481* PCR ruling out the presence of *B. bronchiseptica*, thus suggesting pertussis infection in 26.1 % cases. A random selection of 153 IS*481* positive samples were further analysed by MLVA. The annual distribution of IS*481* positive samples and the representative samples selected for typing are presented in Table 1.

### Demographic characteristics of the children with confirmed *Bordetella* infection

The distribution of the 300 IS*481* positive infants and children according to age, gender, ethnicity and vaccination status is shown in Table 2. The gender distribution was almost equal, i.e. 51 % females. Regarding origin, 50 % were Greek, 31 % were Roma and 19 % were immigrants. With respect to age, the majority were younger than 1 year, while 12 % of the children were older (>1–14 years old). Based on the national vaccination scheme, a complete vaccination status was defined as having received two doses between 3–5 months, three doses between 6–11 months, four doses between 1–4 years and a fifth dose given between 4–6 years.

**Table 2.** Infants and children tested IS*481* positive by PCR during the period of 2010–2015 at ‘Aghia Sophia’ Children’s Hospital, Athens, Greece, and their vaccination status at the time of infection

Age	Gender		Origin				Vaccination status		Total	
	M	F	GR	R	IM	No vaccine	Complete	Incomplete	145	
0–2 m	71	74	86	27	32	145	0	0	145
3–5 m	46	49	41	35	19	61: 10 GR 16 IM 35 R	0	34: 31 GR 3 IM	95
6–11 m	17	7	5	18	1	19: 1 GR 1 IM 17 R	2: 2 GR	3: 2 GR*^a^* 1 R*^b^*	24
1–4 y	6	16	8	11	3	13: 1 GR 1 IM 11 R	8: 7 GR 1 IM	1: 1 IM*^c^*	22
5–14 y	6	8	11	1	2	2: 1 IM 1 R	12: 11 GR 1 IM	0	14

M, male; F, female; GR, Greek; R, Roma; IM, immigrant; m, month; y, years.

Complete^*^=according to age and the national immunization schedule (3–5m=two doses, 6–11 m=three doses, 1–4y=four doses, 5–14y=five doses).

*a,* 1 GR had one dose, 1 GR had two doses; *b,* one dose; *c,* three doses.

### *B. pertussis* molecular types present in Greece

We used MLVA to type the 153 IS*481* positive samples collected in Greece during 2010–2015. A complete MLVA profile was obtained for 43 % of the samples (66/153), 37 % of the samples yielded a partial MLVA profile (57/153), whereas 20 % of the samples yielded no MLVA profile (30/153).

Samples resulting in partial or no MLVA profiles were subjected to SSI diagnostic PCR. Out of 57 samples giving a partial MLVA profile, we confirmed *B. pertussis* DNA in 23 samples, in 24 samples we detected IS*481* DNA, and in 10 samples we were unable to confirm the presence of *Bordetella* spp.*/B. pertussis* DNA. For samples not providing a MLVA profile (30), we confirmed *B. pertussis* DNA in four samples and IS*481* DNA in 14 samples; in 12 samples, we could not detect *Bordetella* DNA. The IS*1001* target was not detected in any of the samples tested using the SSI diagnostic PCR, thus no specimens were found to contain *B. parapertussis/B. bronchiseptica* DNA.

The success rate of obtaining complete MLVA profiles may be correlated to the amount or the quality of DNA in the sample. The amount of DNA in the purified sample was registered by the quantification cycle (C_q_)-value in the diagnostic real-time PCR. The C_q_-value (mean) in samples resulting in a full MLVA profile was 22.4 (C_q_ range 8.6–32.6); the mean C_q_ of samples giving a partial MLVA profile was 33.6 (C_q_ range 28.1–39.7), while the mean C_q_ value of samples not giving a MLVA profile was 35.6 (C_q_ range 31.0–38.7). The quality of the DNA may depend on the handling, storage and shipment of the purified DNA.

**Fig. 1.**
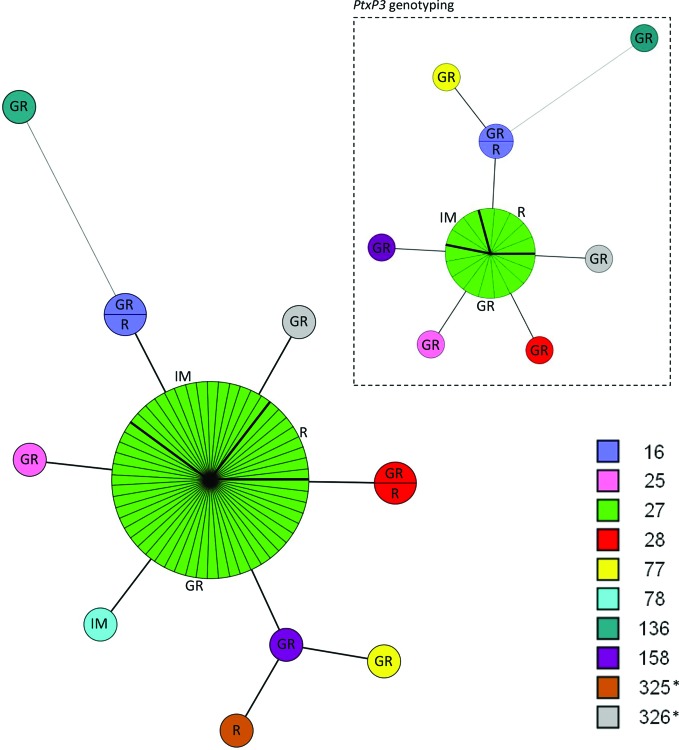
Minimum spanning tree (MST) based on the categorical clustering of multi-locus variable number tandem repeat analysis types (MTs) of 66 *B. pertussis* samples collected in Greece (2010–2015). Each circle within the tree represents a unique MT. Circle size indicates the number of samples in each MT group. The short lines connecting the circles represent single-locus variants. The longer line connecting circles represents a multi-locus (three loci) variant. Each MT has been appointed a unique colour. The coloured boxes to the right denotes the corresponding Dutch type name. *New MTs detected in this study. GR=Greek (40), R=Roma (11), IM=immigrant (15). The green circle representing MT27 is divided into GR, R and IM by bold lines. Insert (top-right corner): MST showing the subset of *B. pertussis* samples (25/66) selected for *ptxP3* genotyping: GR (16), R (6), IM (3). For a few samples harbouring MTs other than MT27 (shown in the primary MST) it was not possible to obtain a PCR product for sequencing of *ptxP* (MT78, MT325).

The 66 complete MLVA profiles were resolved into 10 MTs of which eight were previously described [15] and two were novel. A minimum spanning tree based on the categorical clustering of the 66 MTs is shown in Fig. 1.

The majority of the 66 fully typed samples was resolved into MT27 representing 83 % of the samples. Of the samples with a different MT than MT27, 12 % were single-locus variants compared to MT27 including MT16, MT25, MT28, MT78, MT158 and the novel MT326. Only 3 % of the samples were double-locus variants compared to MT27 comprising MT77 and the novel MT325, whereas MT136 (2 %) was a multi-locus variant from MT27.

The distribution of MTs according to the origin of IS*481* positive children is shown in Fig. 1. *B. pertussis* MT27 was highly represented in infants and children of all origins (33 GR, 8 R and 14 IM); and similarly *B. pertussis* with other MTs were not restricted to infants and children of one origin (7 GR, 3 R and 1 IM). Simpson’s index of diversity (95 % confidence interval) was 0.31 (0.16–0.46) for the total period (2010–2015). The diversity index was also determined for each demographic group of infants and children. The diversity index for Greek infants and children was 0.32 (0.13–0.52). The GR group consisted of 40 samples in total, with 33 samples being MT27 and one sample distributed in each of the following MTs: MT16, MT25, MT28, MT77, MT136, MT158, MT326. The diversity index for Roma infants and children was 0.49 (0.15–0.83). The R group consisted of 11 samples in total with eight being MT27 and one sample distributed on each of the following MTs: MT16, MT28, MT32. The diversity index for immigrant infants and children was 0.13 (1.00–0.36). The IM group consisted of 15 samples in total, with 14 samples being MT27 and one sample being MT78. Based on the 95 % confidence intervals, the genetic diversity of the three demographic groups are not significantly different.

### Pertussis toxin promoter gene (*ptxP*) typing

Among the 66 samples yielding a complete MLVA profile, 25 samples were selected for further analysis. The selection of samples was based on varying MTs as well as on varying ethnical background.

The distribution of samples with respect to MT and ethnical background is shown in Fig. 1 (insert). The samples covered 16 infants and children with Greek background, six with Roma background and three with immigrant background. Furthermore, MT16, MT25, MT27, MT28, MT77, MT136, MT158 and MT326 were covered. Sequencing of MT78 and MT325 was unsuccessful. The 25 sequence-typed samples all contained the *ptxP3* allele.

## Discussion

Molecular typing of IS*481* positive/*B. pertussis* was performed on samples from hospitalized infants and children at the ‘Aghia Sophia’ Children's Hospital, Athens, during the period 2010–2015, providing data for the genetic background of circulating strains in Greece.

When comparing the national vaccination coverage (90 %) in Greece for the first five doses of *B. pertussis* immunization with the vaccination coverage of the study population, we found a very low vaccination coverage in the study population. The vaccination coverage of the hospitalized infants (0–11 months) in the present study includes 2/264 infants with complete coverage and 37/264 with incomplete coverage. For children in the present study (1–14 years), 20/36 were fully covered while 1/36 was incompletely covered. About half of the study population were of Roma or immigrant origin (149 out of 300 infants and children). One Roma child was incompletely immunized whereas the rest were unvaccinated indicating that this migrant population is vulnerable to infection.

Laboratory confirmation of pertussis infection was found in only 1.7 % of the infants (**>**2 months–1 year of age) who were fully vaccinated, indicating the importance of immunization for protection. For fully immunized children who had breakthrough disease, it is unclear whether the disease was due to vaccination failure or due to other immune deficiencies.

A complete MLVA profile was obtained in 43 % (66/153) of the clinical samples in this study. Similar findings have been presented in other studies. A complete MLVA profile was obtained for 52 % of the samples (26/50), when testing clinical specimens originating from Philippine patients in the period 2012–2014 [23], and a complete MLVA profile was obtained in 44 % (34/77) of the samples originating from Austrian patients in the period 2002–2006 [30].

Partial MLVA profiles (obtained in 37.3 % of the samples) may be due to the lack of VNTR loci or mutation in the priming sites, but it is most likely due to the quantity or the quality of available template DNA. This is supported by the C_q_-values from the diagnostic real-time PCR that roughly indicate the level of DNA present in the sample. In addition, the sensitivity of the MLVA typing PCR assay differs from the sensitivity of the diagnostic PCR assay. *B. pertussis* contains a high density of the IS*481* element which is present in 238 copies in the Tohama I reference strain, comprising 6.2 % of the genome [31]. In contrast, the individual VNTRs and the *ptxP* allele are single copy number targets [13]. Hence, a relatively higher concentration of DNA is needed to detect the individual VNTRs and, e.g. the *ptxP* allele than the IS*481* element.

### MT27 is the dominant MLVA-type

The majority of the 66 completely typed samples were resolved into MT27 representing 83 % of all samples. The genetic diversity (Simpson’s index of diversity) in the Greek *B. pertussis* strain population (2010–2015) based on MLVA was 0.31. This number represents a very low genetic diversity compared to the Czech Republic (GD=0.49) (2008–2015) [18] and Austrian (GD=0.56), Swedish (GD=0.56), Dutch (GD=0.52), French (GD=0.51), German (GD=0.62) and Finnish (GD=0.80) genetic diversities calculated for the period 1998–2001 [32]. In Denmark, the genetic diversity was found to be 0.57 in the period from 1997 until 2010, which was immediately after the introduction of the acellular vaccine in 1997 [16]. The dominance of MT27 and the low genetic diversity observed in this study may reflect that typing of *B. pertussis* was performed on samples collected 13–18 years after introduction of the acellular vaccine in Greece.

The *ptxP* allele did not show any variation as all tested samples harboured the *ptxP3* genotype despite samples covering different MTs and different origins. As all tested samples harbouring MT27 also contained the *ptxP3* allele, our findings correlate with previous European studies where MT27 has been associated with the presence of *ptxP3* [14, 33, 34]. In a study by van Gent *et al*. [17], the prevalence of *ptxP3* was found to increase in 12 European countries from 57 % in the time period 1998–2001 to 97 % in the time period 2007–2012. Furthermore, 97 % of the MT27 isolates in the study also harboured the *ptxP3* allele [17]. A detailed characterization of circulating *B. pertussis* strains in Greece will require sequence typing of polymorphic genes, such as vaccine antigens, the pertussis toxin (*ptx*), filamentous haemagglutinin (*fha*), fimbria (*fim*) and pertactin (*prn*).

The investigated infants and children were of native Greek or Roma origin or they were children of immigrants – presumably born in Greece. Therefore, it was speculated whether the way of living, vaccination coverage and/or immigration could have an impact on the genetic diversity of circulating infectious pertussis strains. The question was whether *B. pertussis* strains isolated from Roma and/or immigrant children would express higher genetic diversity than those isolated from native Greek children. We did indeed observe a higher genetic diversity among *B. pertussis* strains derived from Roma infants and children (0.49) than among strains from Greek (0.32) or immigrant infants and children (0.13); however, the 95 % confidence intervals suggest the difference to be non-significant.

As the number of samples in this study is quite low for statistical confirmation, further studies are needed in order to determine if non-vaccinated Roma infants and children contribute to an increase in the genetic diversity among MTs in Greece. In addition, molecular investigation of the genes coding for the vaccine antigens would reveal polymorphisms in and deletions of these genes; thus addressing the issue of pathogen adaptation as a consequence of vaccine pressure in the Greek pertussis population. In general, the study shows that the MT diversity in Greece is low, reflecting the utilization of acellular pertussis vaccines and a high vaccination coverage.

### Conclusions

The genetic diversity of *B. pertussis* in Greece from 2010 to 2015 resembles the concurrent diversity found in other European countries as well as countries across the world using acellular vaccines, with MT27 being the dominant MT in the majority of the fully typed samples. Most of the samples varying from MT27 were single-locus variants. The gene encoding the toxin promoter *ptxP3* was found in all analysed samples.

This is the first time molecular typing of *B. pertussis* has been performed in Greece, providing important data for future epidemiological studies.
